# Tolerance to Central Hypovolemia Is Greater Following Caffeinated Coffee Consumption in Habituated Users

**DOI:** 10.3389/fphys.2020.00050

**Published:** 2020-02-05

**Authors:** Faith K. Pizzey, Erica Tourula, James Pearson

**Affiliations:** ^1^Department of Biology, University of Colorado Colorado Springs, Colorado Springs, CO, United States; ^2^School of Human Movement and Nutrition Sciences, The University of Queensland, Brisbane, QLD, Australia; ^3^Department of Human Physiology and Nutrition, University of Colorado Colorado Springs, Colorado Springs, CO, United States

**Keywords:** caffeine, coffee, central hypovolemia, lower body negative pressure, blood pressure

## Abstract

We investigated the influence of caffeinated coffee consumption on cardiovascular responses and tolerance to central hypovolemia in individuals habituated to caffeine. Thirteen participants completed three trials, consuming caffeinated coffee, decaffeinated coffee or water before exposure to central hypovolemia via lower body negative pressure (LBNP) to pre syncope. Tolerance to central hypovolemia was quantified as cumulative stress index (CSI: LBNP level multiplied by time; mmHg × min). Prior to the consumption of caffeinated coffee, decaffeinated coffee, and water, heart rate (HR: 62 ± 10, 63 ± 9 and 61 ± 8 BPM, respectively), stroke volume (SV: 103 ± 23, 103 ± 17 and 102 ± 18 mL/beat, respectively), and total peripheral resistance (TPR: 14.2 ± 3.0, 14.0 ± 3.0, and 14.3 ± 2.7 mmHg/L/min, respectively), were not different between trials (all *P* > 0.05). Mean arterial pressure (MAP) increased following consumption of all drinks (Post Drink) (Caffeinated coffee: from 86 ± 8 to 97 ± 7; Decaffeinated coffee: from 88 ± 10 to 94 ± 7; and Water: from 87 ± 10 to 96 ± 6 mmHg; all *P* = 0.0001) but was not different between trials (*P* = 0.247). During LBNP, HR increased (*P* = 0.000) while SV decreased (*P* = 0.000) relative to post drink values and TPR as unchanged (*P* = 0.109). HR, SV, and TPR were not different between trials (all *P* > 0.05). MAP decreased at pre syncope in all trials (60 ± 5, 60 ± 7, and 61 ± 6 mmHg; *P* < 0.001). LBNP tolerance was greater following caffeinated coffee (914 ± 309 mmHg × min) relative to decaffeinated coffee and water (723 ± 336 and 769 ± 337 mmHg × min, respectively, both *P* < 0.05). Tolerance to central hypovolemia was greater following consumption of caffeinated coffee in habituated users.

## Introduction

The impact of caffeine on cardiovascular responses to central hypovolemia has been examined during sub-pre syncopal limited head-up tilt tests in habituated users with conflicting findings (Debrah et al., 1995; Berry et al., 2003). However, the influence of caffeine on tolerance to a central hypovolemic challenge, up to the point of pre syncope, is unknown (Crandall et al., 2019). Arterial blood pressure and vascular resistance increase following caffeine or coffee ingestion in caffeine-naïve individuals (Whitsett et al., 1984; Smits et al., 1985a; Pincomb et al., 1988; Casiglia et al., 1991; Hartley et al., 2004; James and Gregg, 2004). However, in habituated users caffeine consumption results in similar increases sympathetic nerve activity (Corti et al., 2002; Sudano et al., 2005) and circulating catecholamines (Izzo et al., 1983) but the pressor response is somewhat attenuated (Izzo et al., 1983; Astrup et al., 1990; Sudano et al., 2005). Understanding the effect of caffeine on cardiovascular responses and tolerance to central hypovolemia in habituated individuals is important given its prevalence.

Coffee is the second most consumed beverage worldwide, behind water (Cappelletti et al., 2015) and the most common form of caffeine ingestion (Frary et al., 2005; Fulgoni et al., 2015). Approximately 90% of Americans report consuming caffeine daily, with the average intake approximately 185 mg per day (Fulgoni et al., 2015). Taken together, this suggests that a considerable percentage of the population are habituated to caffeinated coffee. Furthermore, individuals at an increased risk of experiencing central hypovolemia via a hemorrhagic insult, such as soldiers, have a daily caffeine intake of approximately 200 mg per day, primarily via caffeinated coffee consumption (Lieberman et al., 2012; Knapik et al., 2016). Given this, the aim of this study was to examine the influence of caffeinated coffee on the cardiovascular responses and tolerance to central hypovolemia in habituated users. We studied the effect of caffeinated coffee upon tolerance to central hypovolemia relative to water and decaffeinated coffee to account for increases in arterial blood pressure following fluid consumption (Smits et al., 1985b; Schroeder et al., 2002; Callegaro et al., 2007). We hypothesized that the consumption of caffeinated coffee would prolong tolerance to progressive central hypovolemia in habituated individuals.

## Materials and Methods

Thirteen participants (six females, seven males; age, 26 ± 6 years; height, 174 ± 11 cm; weight, 71.8 ± 14.3 kg and urine specific gravity, 1.017 ± 0.008) participated in this three-visit study in laboratory conditions of 21.8 ± 1.1°C temperature, 26 ± 14% relative humidity and 606 ± 3 mmHg barometric pressure. Participants were recruited via flyers and word of mouth from the general Colorado Springs area. Female subjects were not taking birth control medication and were tested 5 ± 3 days after the onset of menses in order to control for the potential influence of variations in sex hormone concentration throughout the menstrual cycle on neural and hemodynamic responses to orthostatic stress (Usselman et al., 2016), although recent evidence suggests that cardiovascular responses and tolerance to central hypovolemia are not affected by menstrual cycle phase (Convertino et al., 2019). For a detailed review of the effects of sex hormones and the menstrual cycle upon hemodynamic responses and time to presyncope, the reader is referred to Goswami et al. (2019). To be included in the study participants were required to be free of cardiovascular, metabolic and neurologically acting medications and diseases, be non-smokers and regular caffeine consumers. Subjects must also have refrained from alcohol and caffeine for at least 12 h and exercise for at least 24 h before each study visit. Subjects were informed of the purpose, procedures, and risks of the study before providing their informed written consent. The protocol and consent were approved by the institutional review board at the University of Colorado Colorado Springs (IRB 18-075). The study conformed to the standards set by the latest revision of the Declaration of Helsinki.

### Pre-study Information and Controls

On the first study visit, subjects filled out the DSM-V dependency questionnaire to allow researchers to establish a history of caffeine habituation (Cappelletti et al., 2015). Of the twelve criteria contained in the DSM-V, each participant displayed at least two and up to eight criteria (3.8 ± 2.0 criteria) indicating habituation and dependency on caffeine. Prior to each study visit subjects were asked to provide a 5-day diary detailing food and beverages consumed in the 5 days prior to a study visit. This was completed to confirm caffeine dependence and continued habituation prior to each trial, as well as serving as a guide to participants to replicate the meals consumed 12 h before each study visit, in an attempt to ensure similar rates of fluid absorption and caffeine metabolism (Murray, 1987; Brachtel and Richter, 1988).

### Instrumentation and Experimental Protocol

On experimental days, nude body mass was recorded (EB9380H Etekcity, Shenzhen, China) post void. Height was measured using a stadiometer. Arterial blood pressure was continuously measured non-invasively from a finger on the right hand using photoplethysmography (Nexfin, BMEYE, Amsterdam, Netherlands), which was adjusted to heart level using a hydrostatic column and used to calculate mean arterial pressure (MAP) and estimate stroke volume (SV) using pulse contour analysis (Bogert et al., 2010). Photoplethysmography blood pressure readings were confirmed from blood pressure measured by automated auscultation at the level of the brachial artery (5 Series, Omron Healthcare, Kyoto, Japan). Heart rate (HR) was obtained from three-lead electrocardiogram interfaced with bio amp (Lab Chart, ADInstruments, Colorado Springs, CO, United States). Mean skin temperature was measured from the weighted average temperature across six sites (upper and lower back, chest, abdomen, thigh, and calf) (Taylor et al., 1989) using thermocouples (Omega, Connecticut, United States) fixed to the skin with porous adhesive tape. Participants were also instrumented for measurement of skin blood flow on the dorsal right forearm via laser Doppler combined with local heating unit (PF 457; Perimed, North Royalton, OH, United States) connected to a laser-Doppler flowmeter (PF 5010; Perimed). A small diameter thermocouple (Omega, Connecticut, United States) was fixed underneath the laser Doppler and local heating unit to measure local forearm skin temperature at the site of skin blood flow measurement. This measurement was made to ensure similar skin temperatures at the site of the skin blood flow measurement relative to whole body mean skin temperatures. This was achieved by adjusting the temperature of the local heating unit (PF 5020; Perimed, North Royalton, OH, United States). Therefore, skin blood flow measured at the forearm was subject to similar locally and neurally mediated cutaneous vasodilation as the rest of the skin surface area. Skin blood flow was expressed as cutaneous vascular resistance (CVR; mmHg/AU) as calculated from the ratio of MAP (mmHg) to skin blood flow (AU). Following instrumentation, participants rested in the supine position for at least 30 min to allow for the stabilization of fluid shifts and baseline data were then collected.

Participants then consumed either caffeinated coffee, decaffeinated coffee or water in a double-blind randomized study design. All participants were given the beverage to drink in the same stainless steel closed top container which was prepared in a different room. Participants were aware that they would be given one of the three drinks on each study visit but were not told which drink they had been given at any point during the study. Caffeinated and decaffeinated coffee (Nescafè Clàsico, Vevey, Switzerland) and water were all given at the same relative volume (6.25 mL/kg body mass or 455.6 ± 92.5 mL) and temperature (40.2 ± 0.8°C) to control for the influence of fluid temperature on vascular resistance (Drettner, 1964). Caffeine content of the caffeinated coffee trial was calculated from HPLC analysis of the same brand and type of coffee used in this study (Hodgson et al., 2013), allowing calculation of the caffeine content based on the weight of coffee powder. Consequently, 4 mg/kg body mass or 291.4 ± 59.5 mg of caffeine were consumed in the caffeinated coffee trial. Participants then voided their bladder (if necessary) and returned to the supine position where they rested in a supine position for at least 30 min before any further measurements were made. Sixty minutes after the ingestion of the drink participants were moved into the lower body negative pressure (LBNP) chamber, the top of which was sealed at the level of the iliac crest while they lay supine. A neoprene skirting connected to the LBNP chamber was secured around the waist with a belt to ensure an airtight seal. In all trials, participants then underwent an LBNP tolerance test to pre syncope. Before the onset of LBNP, baseline measurements were repeated (Post Drink/pre LBNP) approximately 80 min following the ingestion of the drink (Caffeinated Coffee: 83 ± 16, Decaffeinated Coffee: 83 ± 14, and Water: 83 ± 9 min, *P* = 0.988). LBNP then began at −20 mmHg for 3 min, followed by increments of −10 mmHg in 3 min stages until reaching pre syncope. Photoplethysmography blood pressure readings and derived cardiovascular measurements were made continuously throughout LBNP. Auscultated blood pressure measurements at the brachial artery were made at 3 min intervals at the end of each stage of LBNP, and frequently toward the end of the LBNP test. The termination of LBNP at pre syncope was based on a rapid and progressive decrease in blood pressure resulting in sustained systolic blood pressure <80 mmHg, relative and pronounced bradycardia and/or subject self-reporting pre syncopal symptoms such as persistent nausea or lightheadedness. At the point of pre syncope, the negative pressure inside the LBNP chamber as well as the time taken to reach pre syncope were recorded. Tolerance to LBNP was quantified using the cumulative stress index (CSI) calculated by summing the time at each level of LBNP multiplied by that level (i.e., 20 mmHg × 3 min + 30 mmHg × 3 min + 40 mmHg × 3 min, etc.) until pre-syncope. For example, if an individual reached the point of pre syncope at 16 min 30 s (−70 mmHg LBNP) the maximal CSI is 705 mmHg × min.

### Data Analysis

Data were collected via a data-acquisition system (ADInstruments Colorado Springs, CO, United States). Cardiac output (Q; L/min) was calculated as the product of heart rate (BPM) multiplied by SV (mL/beat). Total peripheral resistance (TPR; mmHg/L/min) was calculated as the MAP (mmHg) divided by cardiac output. Data were averaged across 30 s at baseline (Pre Drink), following the ingestion of the drink prior to LBNP (Post Drink) and throughout the duration of LBNP. Throughout LBNP data were averaged across a 30 s time period, 15 s before and after the time at which 20, 40, 60, and 80% of the maximal CSI occurred, and 5 s immediately prior to pre syncope. All calculations were performed off-line and data were statistically analyzed using a two-way analysis of variance with repeated measures, with main factors of trial (levels: Caffeinated coffee, Decaffeinated coffee, and Water) and time (levels: Pre Drink, Post Drink, 20, 40, 60, and 80% of the CSI, and pre-syncope). *Post hoc* analyses were performed using paired *t*-tests with a Bonferroni correction when a significant main effect or interaction was identified. USG, body mass and CSI were compared between trials using a one-way ANOVA (Main Effect: Trial) and followed up using a paired *t*-test where necessary. Data are reported as mean ± SD.

## Results

**FIGURE 1:**
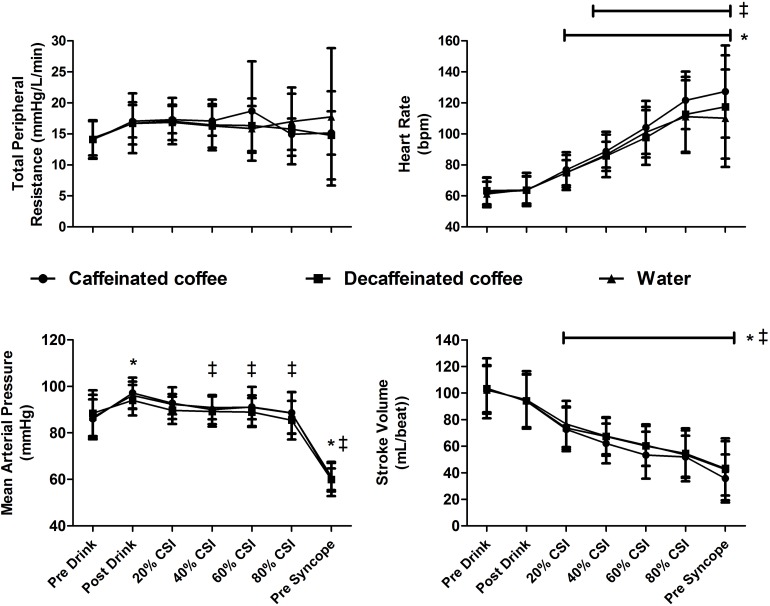
Cardiovascular responses to central hypovolemia. Stroke volume (SV), heart rate (HR), total peripheral resistance (TPR), and mean arterial pressure (MAP) responses are displayed at baseline (Pre Drink), after consumption of either caffeinated coffee, decaffeinated coffee or water (Post Drink), and throughout lower body negative pressure (LBNP). MAP was increased following consumption of all drinks and declined in all trials at pre syncope. HR increased and SV decreased in all trials throughout LBNP relative to pre drink values. Data are displayed as mean ± standard deviation for thirteen participants. *different from baseline in all trials (*P* < 0.05). ‡ different from Post Drink values in all trials (*P* < 0.05).

At baseline, body mass (71.8 ± 14.5, 71.6 ± 14.7, and 72.0 ± 14.7 Kg, *P* = 0.99) and USG (1.015 ± 0.009, 1.020 ± 0.006, and 1.016 ± 0.008, *P* = 0.22) were not different between caffeinated coffee, decaffeinated coffee, and water trials, respectively. At baseline, Pre Drink, MAP (86 ± 8, 88 ± 10, and 87 ± 10 mmHg; Figure 1), HR (62 ± 10, 63 ± 9, and 61 ± 8 BPM), SV (103 ± 23, 103 ± 17, and 102 ± 18 mL/beat), and TPR. MAP, HR, SV, and TPR were not different between trials at any point (Main effect for trial: *P* = 0.247, *P* = 0.127, *P* = 0.324, and *P* = 0.738; respectively).

Mean arterial pressure was different over time in all trials (main effect of time *P* = 0.0001). MAP increased relative to Pre Drink following consumption of caffeinated coffee, decaffeinated coffee, and water (Post Drink: 97 ± 7, 94 ± 7, and 96 ± 6 mmHg, respectively, *P* = 0.0001; Figure 1). TPR tended to increase slightly following drink consumption (Post Drink; Caffeinated: 17.0 ± 2.6; Decaffeinated: 16.7 ± 4.8 and Water 16.7 ± 4.8 mmHg/L/min), but this slight increase was not significantly different (Main effect of time *P* = 0.109). HR was not different at Post Drink relative to Pre Drink in the caffeinated coffee (64 ± 9 BPM), decaffeinated coffee (64 ± 9 BPM) or water trials (64 ± 11 BPM, all *P* > 0.05; Figure 1). Similarly, at Post Drink, SV, cardiac output and CVR were all unchanged relative to Pre Drink (all *P* > 0.05; Table 1). Mean skin temperature increased slightly following the ingestion of all drinks relative to Pre Drink (Main effect of time, *P* = 0.007) but was not different between trials at any time point (all *P* = 0.125, Table 1). CVRi was not different over time (*P* = 0.394) but was lower in the decaffeinated coffee trial relative to both water (*P* = 0.003) and caffeinated coffee trials (*P* = 0.001, Table 1).

**TABLE 1:** Cardiovascular and skin temperature data in caffeinated coffee, decaffeinated coffee and water trials.

	**Pre drink**			**Post drink**			**20% CSI**			**40% CSI**			**60% CSI**			**80% CSI**			**Pre syncope**		
**Trial**	**Caff**	**Decaff**	**Water**	**Caff**	**Decaff**	**Water**	**Caff**	**Decaff**	**Water**	**Caff**	**Decaff**	**Water**	**Caff**	**Decaff**	**Water**	**Caff**	**Decaff**	**Water**	**Caff**	**Decaff**	**Water**
**Cutaneous Vascular Resistance (mmHg/AU)**	5.1 ± 2.6	4.2 ± 2.2	4.5 ± 3.7	4.6 ± 2.4	2.8 ± 0.9	4.1 ± 2.5	4.3 ± 2.4	2.9 ± 1.0	4.1 ± 2.6	4.5 ± 2.3	3.2 ± 1.2	4.2 ± 2.8	4.9 ± 2.4	3.3 ± 1.1	4.4 ± 2.7	5.4 ± 2.7	3.4 ± 1.0	4.9 ± 3.0	4.6 ± 1.8	3.8 ± 2.4	5.5 ± 2.9
**Mean Skin Temperature (°C)**	34.1 ± 0.6	33.9 ± 0.8	33.9 ± 0.9	34.6 ± 0.7*	34.5 ± 0.7*	34.5 ± 0.6*	34.5 ± 0.7*	34.5 ± 0.7*	34.4 ± 0.6*	34.5 ± 0.6*	34.4 ± 0.7*	34.4 ± 0.6*	34.5 ± 0.6*	34.4 ± 0.7*	34.3 ± 0.7*	34.5 ± 0.6	34.3 ± 0.6	34.2 ± 0.7	34.5 ± 0.6	34.4 ± 0.7	34.2 ± 0.7
**Cardiac Output (L/min)**	6.3 ± 1.1	6.5 ± 1.3	6.2 ± 1.1	5.8 ± 1.0	6.0 ± 1.3	6.0 ± 1.1	5.5 ± 0.9	5.4 ± 0.9	5.6 ± 0.9	5.4 ± 0.9	5.7 ± 1.0	5.8 ± 1.1	5.4 ± 1.5	5.7 ± 1.1	6.0 ± 1.1	6.1 ± 1.2	5.9 ± 1.4	5.6 ± 1.3	4.1 ± 0.9*‡	4.5 ± 1.2*‡	4.2 ± 1.5*‡

Caff, caffeinated coffee. Decaff, decaffeinated coffee. Data are mean ± standard deviation from 13 participants. *Different from Pre Drink within trials (all *P* < 0.05). ‡Different from Post Drink within trials (all *P* < 0.05).

**FIGURE 2:**
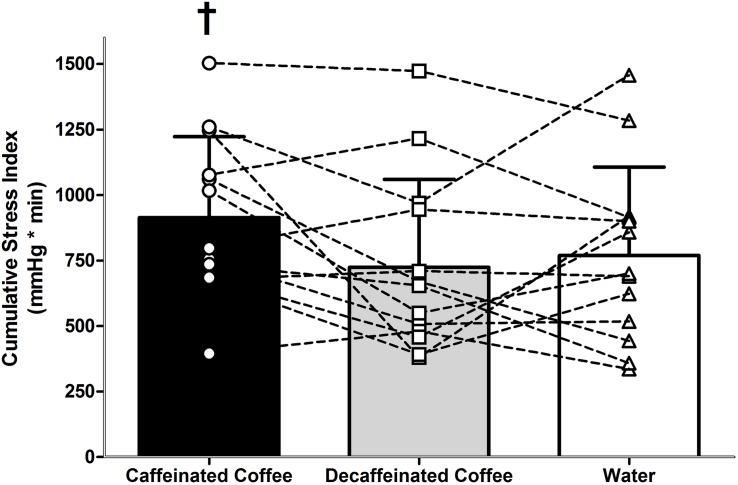
Tolerance to progressive central hypovolemia, via lower body negative pressure, as expressed as cumulative stress index (CSI). Tolerance to central hypovolemia was greater in the caffeinated coffee trial relative to water (*P* = 0.048) and decaffeinated coffee (*P* = 0.033). Data are displayed in bars represent mean ± standard deviations for thirteen participants. Data displayed overlaid onto bars as squares, circles, and triangles represent individual CSI values for each of the thirteen participants in the caffeinated coffee, decaffeinated coffee, and water trials, respectively. † different from decaffeinated coffee and water trials (both *P* < 0.05).

Heart rate and SV were different during LBNP in all trials (main effect of time, both *P* = 0.001). During LBNP, HR increased while SV decreased in all trials relative to both Pre Drink and Post Drink values (Figure 1, all *P* < 0.05). Cardiac output was maintained throughout LBNP up until the point of pre syncope where it decreased in all trials relative to Pre Drink and Post Drink values (*P* < 0.05; Table 1). MAP was decreased at the point of pre syncope relative to Pre Drink and Post Drink (caffeinated coffee: 60 ± 5, decaffeinated coffee: 60 ± 7, and Water: 61 ± 6 mmHg; all *P* < 0.001; Figure 1). CSI was different between trials (Main effect: *P* = 0.048; Figure 2) and greater following ingestion of caffeinated coffee (914 ± 309 mmHg × min) relative to the decaffeinated coffee (723 ± 336 mmHg × min; *P* = 0.033) and water trials (769 ± 337 mmHg × min, *P* = 0.048). CSI was not different between decaffeinated coffee and water trials (*P* = 0.581). In seven participants, CSI was highest in the caffeinated coffee trial. In the other six participants, CSI was highest in either the decaffeinated coffee (*n* = 4) or water (*n* = 2) trials (Figure 2).

## Discussion

Hemorrhage is a leading cause of mortality following trauma in the general population (Kauvar et al., 2006) and a major form of potentially survivable death on the battlefield (Eastridge et al., 2011). Caffeinated coffee is consumed on a daily basis by the majority of the United States population (Frary et al., 2005; Cappelletti et al., 2015; Fulgoni et al., 2015) and individuals with an increased risk of experiencing central hypovolemia following a hemorrhagic injury, such as soldiers (Lieberman et al., 2012; Knapik et al., 2016). However, the influence of caffeine on cardiovascular responses and tolerance to a central hypovolemic challenge is unknown (Crandall et al., 2019). Therefore, the aim of this study was to examine the influence of caffeinated coffee on the cardiovascular responses and tolerance to central hypovolemia in habituated users. The findings of this study indicate that tolerance to central hypovolemia was slightly greater following consumption of caffeinated coffee relative to decaffeinated coffee and water.

In habitual consumers, caffeine or coffee ingestion increases sympathetic nerve activity (Corti et al., 2002; Sudano et al., 2005), circulating catecholamines (Izzo et al., 1983), and arterial blood pressure (Izzo et al., 1983; Astrup et al., 1990; Sudano et al., 2005). This is presumably due to an increase in vascular resistance and/or SV (Pincomb et al., 1985; Hartley et al., 2004), given that HR does not increase in habitual consumers (Izzo et al., 1983; Astrup et al., 1990; Corti et al., 2002; Sudano et al., 2005). In line with this, MAP was similarly elevated following consumption of caffeinated coffee, decaffeinated coffee and water (Figure 1). This pressor response following drink consumption likely owed to a small, but not statistically significant, increase in TPR (+3 mmHg/L/min, *P* = 0.187) as SV (*P* = 0.338) and HR (*P* = 1.0) did not increase relative to pre drink, baseline values (Figure 1). However, the greater LBNP tolerance in the caffeinated coffee trial cannot be attributed to a greater pressor effect prior to LBNP as this was not different between trials.

Mean arterial pressure was reduced similarly in all trials at pre syncope and was not different between trials at any point during LBNP despite a greater LBNP tolerance in the caffeinated coffee trial (Figure 1). Although we were unable to measure central blood volume in this study, this suggests that MAP was maintained for longer, presumably in the presence of a greater central hypovolemia, in the caffeinated coffee trial. Increases in HR and TPR are an important component of the compensatory response to maintain blood pressure during progressive central hypovolemia (Convertino and Sather, 2000; Lackner et al., 2010; Convertino, 2012; Goswami et al., 2015; Patel et al., 2016; Xiang et al., 2018). However, TPR and CVR were unchanged during LBNP in all trials (Table 1). Prior to LBNP, TPR slightly increased following the consumption of all drinks (Pre Drink: ∼14 mmHg/L/min to Post Drink: ∼17 mmHg/L/min, Figure 1) but this was not significantly different (*P* = 0.109). While speculative, the absence of an increase in TPR during LBNP may at least in part owe to the increased arterial blood pressure that occurred following drink consumption and prior to LBNP in all trials (Figure 1).

Given that TPR was unchanged and SV decreased in all trials throughout LBNP (Figure 1) suggests that increases in HR were of particular importance to the maintenance of blood pressure during central hypovolemia. During LBNP, HR increased similarly in all trials indicating that the cardiovascular responses to central hypovolemia were not different between trials at the same relative severity of LBNP, expressed here as percentage of maximal CSI. However, LBNP tolerance and CSI were greater in the caffeinated coffee trial. Therefore, the same relative CSI between trials corresponds to a greater absolute CSI in the caffeinated coffee trial. This suggests that arterial blood pressure could be maintained at a similar cardiovascular strain despite a higher absolute CSI in the caffeinated coffee trial. For example, at 60% of the CSI, HR was not different between trials despite a greater absolute CSI in the caffeinated coffee trial (HR: 104 ± 17 BPM, CSI: 539 mmHg × min), relative to the decaffeinated coffee and water trials (HR: 98 ± 18 BPM, and 101 ± 16 BPM, CSI: 434 and 442 mmHg × min, respectively). This is suggestive of an increased cardiovascular reserve to maintain arterial blood pressure with progressive central hypovolemia, thereby contributing to the increased tolerance in the caffeinated coffee trial.

Caffeine consumption increases sympathetic nerve activity in habituated users, while HR is either unchanged or slightly reduced (Corti et al., 2002; Sudano et al., 2005). This may owe to a baroreflex mediated inhibition of cardiac sympathetic activity that is secondary to increases in arterial blood pressure following coffee consumption (Corti et al., 2002). The increase in MAP in response to caffeinated coffee consumption was not different relative to decaffeinated coffee or water. Therefore, any inhibition of sympathetic drive was likely to be similar between trials. However, any baroreflex mediated sympathetic inhibition likely subsided during the latter stages of LBNP (>40% CSI; Figure 1) when cardiopulmonary and arterial baroreceptor unloading were more pronounced and MAP was reduced relative to post drink values (Figure 1). In the caffeinated coffee trial this occurred at the same relative but greater absolute CSI and may have been associated with a greater increase in sympathetic drive relative to decaffeinated coffee and water. For example, while not statistically different between trials, HR was approximately 10 beats per minute higher in the caffeinated coffee trial both at 80% of the CSI and at pre syncope, relative to decaffeinated coffee and water trials. This may have enabled a better maintenance of arterial blood pressure during progressive central hypovolemia thereby contributing to the increased tolerance relative to decaffeinated coffee and water trials.

The influence of caffeinated coffee upon tolerance to central hypovolemia was variable between subjects. Seven out of thirteen participants in this study demonstrated the highest tolerance to central hypovolemia in the caffeinated coffee trial (Figure 2). Therefore, while these data indicate a statistically greater tolerance to central hypovolemia following consumption of caffeinated coffee, this effect was variable between participants. It is beyond the scope and sample size of the present study to identify the reasons for this apparent individual variation in the response to central hypovolemia between trials. However, five out of the seven participants who demonstrated the highest tolerance to central hypovolemia following caffeinated coffee consumption were male. This lends to the possibility that the impact of caffeinated coffee upon the underlying cardiovascular responses, and tolerance to central hypovolemia may be different between males and females.

### Perspectives and Significance

Caffeinated coffee is consumed daily by the majority of the United States population and is the second most commonly consumed beverage worldwide behind water (Frary et al., 2005; Cappelletti et al., 2015; Fulgoni et al., 2015). This suggests that a considerable percentage of the world population are habituated to caffeinated coffee consumption. Understanding the influence of caffeinated coffee on cardiovascular responses and tolerance to central hypovolemia up to the point of pre syncope is important for populations exposed to an increased risk of experiencing a hemorrhagic insult. Soldiers, for example, consume approximately 200 mg of caffeine per day, primarily in caffeinated coffee (Lieberman et al., 2012; Knapik et al., 2016). Tolerance to central hypovolemia was greater following consumption of caffeinated coffee although the difference relative to water and decaffeinated coffee was modest (Δ + 145 and + 191 mmHg × min, respectively). A modest increase in tolerance to central hypovolemia following caffeinated coffee consumption in a habituated population is important to understand given that hemorrhage is a leading cause of mortality following trauma in the general population (Kauvar et al., 2006) and a major form of potentially survivable death on the battlefield (Eastridge et al., 2011). Further studies are warranted to better understand the mechanisms underlying this increased tolerance to central hypovolemia with caffeinated coffee consumption, particularly given the aforementioned variability between participants (Figure 2). Such studies may lead to a better understanding of cardiovascular control during, and tolerance to progressive central hypovolemia in individuals who are both habituated to caffeine and at increased risk of experiencing a hemorrhagic insult, such as soldiers.

### Limitations

First, nine of thirteen subjects (∼70%) correctly identified caffeinated and decaffeinated coffee trials. While this speaks to the habituation of participants, we are unable to rule out a placebo effect during these trials. A taste control placebo was provided in the form of the decaffeinated coffee, yet caffeine elicits a cephalic-cardiac response which would be almost absent in decaffeinated coffee (Mcmullen et al., 2012). Participants also identified as being habituated to caffeinated coffee consumption and dependent on caffeine as per the DSM-V. In these settings, it is difficult to provide a true placebo for the perceived effects of caffeinated coffee, given that participants were familiar with any physiological effect they habitually experience following caffeine consumption. Nevertheless, it is possible that a placebo effect contributed to the modest increase in LBNP tolerance observed in the caffeinated coffee trial. Second, we measured cardiovascular responses to caffeinated coffee approximately 80 min after ingestion and tolerance to central hypovolemia thereafter. We designed the study in this way in order to measure cardiovascular responses to caffeinated coffee and tolerance to LBNP within the time taken to reach peak plasma caffeine concentrations [15–120 min (Medicine, 2001)]. We chose this time frame as the peak caffeine concentration in plasma has been shown to be just under 60 min in a caffeine-habituated population following consumption of a similar volume of hot coffee to that administered here (White et al., 2016). Therefore, it is reasonable to suggest that caffeine concentrations in plasma at the time of the post drink measurement were considerably elevated during LBNP. However, it is possible that the findings of this study may be different had we waited for a longer period of time after the ingestion of caffeinated coffee before measuring cardiovascular responses and tolerance to central hypovolemia. Third, the compensatory reserve available to protect arterial blood pressure during central hypovolemia is influenced by blood volume (Stewart et al., 2014; Gagnon et al., 2016). We did not measure an index of circulating blood volume. Therefore, we are unable to determine whether one of the consumed drinks had a larger effect upon blood volume and LBNP tolerance that another. However, body mass and USG were measured before each trial and were not different between trials which suggests that each participant was likely to at least have begun each study visit at a similar hydration status and presumably circulating blood volume.

## Conclusion

In habitual caffeine consumers, caffeinated coffee a modestly increased tolerance to central hypovolemia. These data have implications for the understanding of cardiovascular responses and tolerance to central hypovolemia in individuals habituated to caffeine, such as the general United States population and individuals such as soldiers who are at an increased risk of hemorrhagic insult.

## Data Availability Statement

The datasets generated for this study are available on request to the corresponding author.

## Ethics Statement

The studies involving human participants were reviewed and approved by the Institutional Review Board University of Colorado Colorado Springs. The patients/participants provided their written informed consent to participate in this study.

## Author Contributions

FP contributed to the study design, collected the data, performed the analysis, and wrote the manuscript. ET collected the data, performed the analysis, and wrote the manuscript. JP conceived and designed the analysis, collected the data, contributed the data and analysis tools, performed the analysis, and wrote the manuscript.

## Conflict of Interest

The authors declare that the research was conducted in the absence of any commercial or financial relationships that could be construed as a potential conflict of interest.
